# Extrinsic and Intrinsic Brain Network Connectivity Maintains Cognition across the Lifespan Despite Accelerated Decay of Regional Brain Activation

**DOI:** 10.1523/JNEUROSCI.2733-15.2016

**Published:** 2016-03-16

**Authors:** Kamen A. Tsvetanov, Richard N.A. Henson, Lorraine K. Tyler, Adeel Razi, Linda Geerligs, Timothy E. Ham, James B. Rowe, Cambridge Centre for Ageing and Neuroscience

**Affiliations:** ^1^Centre for Speech, Language and the Brain, Department of Psychology, University of Cambridge, Cambridge CB23 6HT, United Kingdom,; ^2^Medical Research Council Cognition and Brain Sciences Unit, Cambridge CB2 7EF, United Kingdom,; ^3^The Wellcome Trust Centre for Neuroimaging, University College London, London WC1N 3BG, United Kingdom,; ^4^Department of Electronic Engineering, NED University of Engineering and Technology, Karachi, Pakistan,; ^5^Department of Clinical Neurosciences, Cambridge University, Cambridge CB2 0SP, United Kingdom,; ^6^Behavioural and Clinical Neuroscience Institute, Cambridge CB23 6HT, United Kingdom, and; ^7^Cambridge Centre for Ageing and Neuroscience (Cam-CAN), University of Cambridge and MRC Cognition and Brain Sciences Unit, Cambridge CB2 7EF, United Kingdom

**Keywords:** aging, between-/within-network, cross-spectral dynamic causal modelling, fMRI, resting-state networks, salience network

## Abstract

The maintenance of wellbeing across the lifespan depends on the preservation of cognitive function. We propose that successful cognitive aging is determined by interactions both within and between large-scale functional brain networks. Such connectivity can be estimated from task-free functional magnetic resonance imaging (fMRI), also known as resting-state fMRI (rs-fMRI). However, common correlational methods are confounded by age-related changes in the neurovascular signaling. To estimate network interactions at the neuronal rather than vascular level, we used generative models that specified both the neural interactions and a flexible neurovascular forward model. The networks' parameters were optimized to explain the spectral dynamics of rs-fMRI data in 602 healthy human adults from population-based cohorts who were approximately uniformly distributed between 18 and 88 years (www.cam-can.com). We assessed directed connectivity within and between three key large-scale networks: the salience network, dorsal attention network, and default mode network. We found that age influences connectivity both within and between these networks, over and above the effects on neurovascular coupling. Canonical correlation analysis revealed that the relationship between network connectivity and cognitive function was age-dependent: cognitive performance relied on neural dynamics more strongly in older adults. These effects were driven partly by reduced stability of neural activity within all networks, as expressed by an accelerated decay of neural information. Our findings suggest that the balance of excitatory connectivity between networks, and the stability of intrinsic neural representations within networks, changes with age. The cognitive function of older adults becomes increasingly dependent on these factors.

**SIGNIFICANCE STATEMENT** Maintaining cognitive function is critical to successful aging. To study the neural basis of cognitive function across the lifespan, we studied a large population-based cohort (*n* = 602, 18–88 years), separating neural connectivity from vascular components of fMRI signals. Cognitive ability was influenced by the strength of connection within and between functional brain networks, and this positive relationship increased with age. In older adults, there was more rapid decay of intrinsic neuronal activity in multiple regions of the brain networks, which related to cognitive performance. Our data demonstrate increased reliance on network flexibility to maintain cognitive function, in the presence of more rapid decay of neural activity. These insights will facilitate the development of new strategies to maintain cognitive ability.

## Introduction

The maintenance of wellbeing across the lifespan depends on cognitive function (Nyberg et al., 2012; Sahakian, 2014), although the factors that support cognitive performance in older age are poorly understood. Cognitive performance is associated with communication between brain regions that is intrinsic to large-scale functional networks, as well as extrinsic interactions between such networks (Fox et al., 2005; Kelly et al., 2008). These interactions may change with age (Onoda et al., 2012). We tested the hypothesis that interactions within (intrinsic) and between (extrinsic) large-scale functional networks determine neurocognitive health, and that these interactions are increasingly important for maintaining cognitive function with age.

The functional networks underlying age-related cognitive decline are often studied using functional magnetic resonance imaging (fMRI) during tasks that are related to specific cognitive domains, such as memory, attention, language or executive function (Grady, 2012). Between regional fMRI coactivity has been used as a marker for the presence and strength of brain networks. The same principal task networks can also be identified from interregional correlations in the resting-state fMRI (rs-fMRI) time series (Smith et al., 2009), offering the opportunity to study simultaneously the intrinsic and extrinsic connectivity of multiple functional networks (Cole et al., 2014). The expression of such connectivity changes with age (Ferreira and Busatto, 2013). Furthermore, there is evidence for age-related associations of within-network connectivity and specific cognitive functions (Baldassarre and Corbetta, 2015), as well as between-network interactions and cognitive functions, such as long-term memory (Chan et al., 2014) and working memory (Keller et al., 2015).

However, the fMRI signals used to measure connectivity in these earlier studies cannot disambiguate the vascular and neural components (Logothetis, 2008), which complicates the study of aging (Tsvetanov et al., 2015). Furthermore, defining connectivity in terms of simple correlation between time series does not distinguish the direction of coupling between regions. Dynamic causal modeling (DCM) was developed to address these limitations (Friston et al., 2003), comparing networks in which directed neural influences within and between nodes (regions of interest) are modeled by time-dependent differential equations. This neural activity is mapped to the fMRI signal via a hemodynamic forward model, which separates estimation of the neural coupling parameters from the vascular parameters associated with each node. Importantly, the vascular parameters can differ not only across nodes, but also across individuals, for example as a function of age (Hutchison et al., 2013). If the neural interactions between nodes, as well as the neurovascular coupling, change with age, then the converse is that a participant's age should be predictable by the combination of DCM parameters.

This framework has recently been extended to include rs-fMRI (Friston et al., 2014; Razi et al., 2015), fitting the complex cross-spectral density using a power-law model of the coupled dynamics of neuronal populations, referred to as spectral DCM. The advantage of this simple two-parameter (amplitude and exponent) power-law model is that it renders the generative model deterministic while accommodating stochastic fluctuations in neural states (Friston et al., 2011). This also makes it computationally tractable for large studies. We used spectral DCM to estimate the parameters of models defined by a small number of nodes, where each of these nodes represented a well established functional network associated with cognition in old age (Baldassarre and Corbetta, 2015), namely: the salience network, dorsal attention network, and default mode network. This allowed us to investigate the relationship between age and interactions between these networks, i.e., to test the hypothesis that age alters the influence of the salience network on the other two (Sridharan et al., 2008). Furthermore, we investigated the effect of age within each network, i.e., to determine whether age affects the stability of regional activity as captured by local decay functions (Pinotsis et al., 2013). Most importantly for our overarching hypothesis, we then examined how the effects of age on connectivity relate to cognitive performance. We predicted that interactions within and between large-scale networks are increasingly important for neurocognitive health as we get older.

## Materials and Methods

### 

#### 

##### Participants.

**Figure 1.**
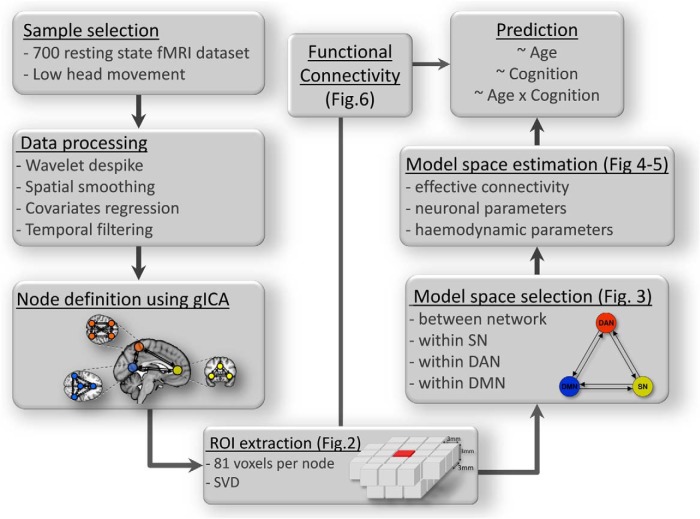
Overview of key processing steps for predictive analysis of age and cognition from rs-fMRI spectral DCM parameters. GICA, group-ICA.

Figure 1 provides a schematic representation of the study and image processing pipeline. A population-based sample of 635 healthy human adults (314 males and 321 females) was collected as part of the Cambridge Centre Aging and Neuroscience (Cam-CAN; Shafto et al., 2014). Ethical approval was obtained from the Cambridgeshire 2 Research Ethics Committee. Participants gave written informed consent. Exclusion criteria included poor vision (<20/50 on Snellen test; Snellen, 1862) and poor hearing (threshold 35 dB at 1000 Hz in both ears), ongoing or serious past drug abuse as assessed by the Drug Abuse Screening Test (DAST-20; Skinner, 1982), significant psychiatric disorder (e.g., schizophrenia, bipolar disorder, personality disorder) or neurological disease (e.g., known stroke, epilepsy, traumatic brain injury); a detailed description of exclusion criteria can be found in Shafto et al. (2014). At an initial home assessment, all participants completed the Mini-Mental State Examination (>25; Folstein et al., 1975). Handedness was assessed using Edinburgh Handedness Inventory (Oldfield, 1971).

Participants performed a battery of cognitive tasks outside the scanner (for a full description, see Shafto et al., 2014). These tests spanned major cognitive domains, speed of processing and intelligence, including: the Cattell culture fair test of fluid intelligence (Cattell and Cattell, 1960), the spot-the word test (Baddeley et al., 1993) as a measure of crystallized intelligence, visual short-term memory as a measure of working memory, motor response consistency (i.e., the inverse of response variability), the Hotel task as a measure of multitasking (Manly et al., 2002), and Benton faces as a measure of face recognition (Benton et al., 1983).

##### MRI acquisition and preprocessing.

Imaging data were acquired using a 3T Siemens TIM Trio. A 3D structural MRI was acquired on each participant using T1-weighted sequence with generalized autocalibrating partially parallel acquisition [(GRAPPA) acceleration factor 2; repetition Time (TR) = 2250 ms; echo time (TE) = 2.99 ms; inversion time (TI) = 900 ms; flip angle α = 9°; field-of-view (FOV) = 256 × 240 × 192 mm; resolution = 1 mm isotropic] with acquisition time of 4 min and 32 s.

For rs-fMRI measurements, echoplanar imaging (EPI) data of 261 volumes were acquired with 32 slices (sequential descending order), slice thickness of 3.7 mm with a slice gap of 20% for whole-brain coverage (TR = 1970 ms; TE = 30 ms; flip angle α = 78°; FOV = 192 × 192 mm; resolution = 3 × 3 × 4.44 mm) during 8 min and 40 s. Participants were instructed to lie still with their eyes closed. The initial six volumes were discarded to allow for T1 equilibration. The imaging data were analyzed using Automatic Analysis (AA 4.0; Cusack et al., 2014) pipelines and modules which called relevant functions from SPM12 (Wellcome Department of Imaging Neuroscience, London, UK). The T1 image was initially coregistered to the MNI template, and the T2 image was then coregistered to the T1 image using a rigid-body transformation. The coregistered T1 and T2 images were used in a multichannel segmentation (SPM12 Segment, based on “New Segment” in SPM8; Ashburner and Friston, 2005) routine to extract probabilistic maps of six tissue classes: GM, WM, CSF, bone, soft tissue, and residual noise. The native-space GM and WM images for all participants (*n* = 635) who passed quality-control checks were then submitted to diffeomorphic registration (Ashburner, 2007) to create group template images (Taylor et al., 2015). To quantify the total motion for each participant, the root mean square volume-to-volume displacement was computed using the approach of Jenkinson et al. (2002). Participants with two or more SD above the group mean motion displacement were excluded from further analysis. This led to the exclusion of 33 participants, i.e., 602 participants included in further analysis. To further ensure this age-related increase in head motion does not affect later analysis of connectivity, we took two further steps: (1) rs-fMRI data were further preprocessed by wavelet despiking, and (2) a subject-specific estimate of head movement (Jenkinson et al., 2002) was included as a covariate in group-level analysis.

The group template was then normalized to the MNI template using a 12-parameter affine transformation. The EPI data were unwarped (using field-map images) to compensate for magnetic field inhomogeneities, realigned to correct for motion, and slice-time corrected to the middle slice. The normalization parameters from the T1 stream were then applied to warp functional images into MNI space. Further processing procedures of the resting-state time series were performed as follows. The normalized images were smoothed (8 mm Gaussian kernel). The first step was to apply data-driven wavelet-despiking approach to minimize motion artifacts (Patel et al., 2014). We observed a high association between the amount of average despiking and head motion across subjects (*r* = 0.740, *p* < 0.001), indicating that the approach accurately identified and corrected for differences in motion artifacts. We also included linear and quadratic detrending of the fMRI signal, covarying out white matter (WM) and CSF signal, and regression of the motion parameters and their first derivatives. WM and CSF signals were estimated for each volume from the mean value of WM and CSF masks derived by thresholding SPM's tissue probability maps at 0.75. The resting data were bandpass filtered (0.008–0.1 Hz).

##### Region-of-interest time-series extraction.

**Figure 2.**
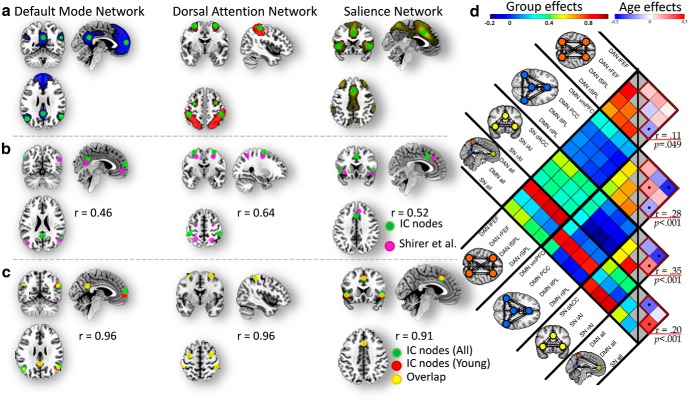
ROI definition using spatial independent component analysis. ***a***, Spatial distribution of three ICs using group ICA (*n* = 602) identified as the DMN (blue), the DAN (red), and SN (yellow), and the peaks of their corresponding nodes (green circles). Spatial correspondence of network nodes identified in (***b***) current study (green) and Shirer et al., 2012 (pink), and (***c***) all participants and young participants (*n* = 100, age 18–40 years). *r* values determine the spatial overlap of the entire networks. ***d***, Temporal correlation (Fisher-*z* transformed *r* values) between SVD time series across all pairs of nodes and networks across all subjects (group effects, left-hand-side correlation map) and their association with age tested in four separate multiple linear regressions (aging effects, right-hand-side correlation map with corresponding *r* and *p* values for each model).Black circles indicate connections contributing strongly (i.e., significant at 5% significance level, FDR corrected) to each model. Gray boxes in the correlation map indicate no values. All refers to SVD of all voxels across all nodes within a given network.

The location of the key cortical regions in each network was identified by spatial ICA, using the Group ICA for fMRI Toolbox (GIFT; http://mialab.mrn.org/software/gift; Calhoun et al., 2001) to extract 20 low-dimensional components (Biswal et al., 2010; Shirer et al., 2012) from the preprocessed rsfMRI data. The three networks were identified by spatially matching to pre-existing templates (Shirer et al., 2012). To ensure that there was no age bias in the selection of the networks, the group independent components (ICs) were further matched to the ICs from a group-ICA in a subgroup of young adults (*n* = 100, age range 18–40; Fig. 2. The salience network (SN) contains three nodes: the dorsal cingulate cortex (dACC), and the right and left anterior insulae (rAI/lAI). The default mode network (DMN) contains four nodes: the ventromedial prefrontal cortex (vmPFC), right and left inferior parietal lobes (rIPL/lIPL), and posterior conjugate cortex (PCC). The dorsal attention network (DAN) contains four nodes: the right and left frontal eye field (rFEF/lFEF), and the right and left superior parietal lobes (rSPL/lSPL).

After identifying the regions-of-interest (ROIs) for each of the networks, we extracted ROI-specific time series from the original preprocessed data for use in the DCM analysis. The ROI time series were defined as the first principal component resulting from the singular value decomposition (SVD) of confound corrected voxels in a (8 mm radius) sphere (Razi et al., 2015), which was centered on the peak voxel for each node (clusters >100 voxels) within each network (group ICs; Fig. 2, green circle). For within-network analysis, we used the ROIs within each respective network. For between network analysis, we used subject-specific ICA time courses for each network (Hyett et al., 2015).

A functional connectivity analysis using Fisher *z*-transformed correlation coefficients among the ROIs' time series was also performed. This indicated that the ROIs were truly representative of the activation within and between networks (Fig. 2).

##### DCM and model space selection.

The spectral DCM analyses were conducted using DCM12 (v6142) implemented in the SPM12 (revision 6225, www.fil.ion.ucl.ac.uk/spm). For each participant, the average effective connectivity between regions across the duration of the resting-state analysis was modeled (i.e., A-matrix). For each analysis, we created a full set of alternate generative models to allow us to explore model space of competing biologically plausible networks, which represent alternate hypotheses of between- and within-network interactions.

To assess the interaction between the three core networks (i.e., extrinsic connectivity), we initially created a full set of models, i.e., every possible mathematical combination of models (*N* = 2^(^*^n^* ^× *n*)^ = 2^(3 × 3)^ = 512, where *N* is the total number networks/nodes and *n* is the number of models, i.e., all possible values for a 3 × 3 matrix, where elements in this matrix can only be either 0 or 1). We then excluded biologically implausible models, as defined by: (1) any network that was not intrinsically connected to itself (i.e., the region did not exert a level of self-inhibition, *N* = 2^(*n* × *n*) − *n*^), and (2) any network that was completely disconnected from every other network. This resulted in the full set of 54 biologically plausible connected models of between-network interactions that is possible with three nodes (data not shown, available on request).

For the characterization of interactions between the three nodes within-SN analysis (dACC, lAI, and rAI), we used the same topology to define the model space as for the between-network analysis (*N* = 54).

For the within-DAN analysis, we further excluded any model that was asymmetrical along the sagittal plane and any model with unilateral connections, either between rFEF and lFEF, or rSPL and lSPL. This resulted in the full set of 13 connected models possible with three nodes.

For the within-DMN analysis, similarly to previous reports (Di and Biswal, 2014), we reduced the model space by excluding any model that was asymmetrical along the sagittal plane or had no direct connection between PCC and vmPFC. This resulted in 30 connected plausible models.

##### Connectivity parameters predicting age and behavioral variability.

**Figure 3.**
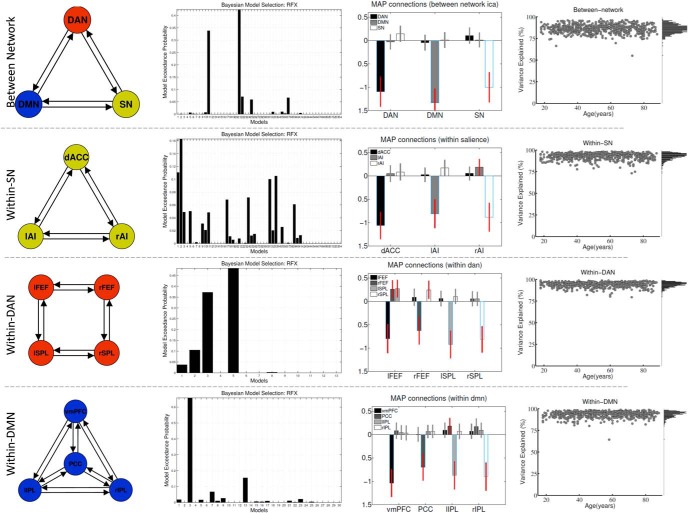
Winning models for between- and within-network analyses. From left to right column, visual representation of the connections between networks/nodes for the full model (Column 1), the exceedance probability (Column 2) for all models, group-averaged connections using model averaging (Column 3), and variance explained, which was age-independent (scatter plots) and was >75% for most subjects (histograms along the right side).

Having estimated the DCM for all models on a participant level, we used Bayesian model selection (BMS; Stephan et al., 2009; Rigoux et al., 2014) to determine the most likely model for the observed dataset, adjusting data fit by model complexity, as defined by the free energy bound on the model evidence (Friston, 2010). BMS was conducted for all participants (*n* = 602) given the high levels of convergence of DCM and variance explained across models and network analysis. The variance explained was >75% in all models for the majority of participants and was age-independent (Fig. 3).

Using Bayesian model averaging (Hoeting et al., 1999; Penny et al., 2010), we asked whether the participant's age and behavioral performance across a range of tasks could be predicted by DCM parameters of the model space. Three sets of parameters were used to predict in a multiple linear regression participants' chronological age. Specifically, (1) all parameters of intrinsic (effective) connectivity (DCM.Ep.A, Friston et al., 2003), (2) values of the amplitude and exponent parameters (DCM.Ep.a) characterizing the power-law distribution of endogenous neural fluctuations (that drive neural populations) assumed by spectral DCM for rs-fMRI (Friston et al., 2014), and (3) values of hemodynamic parameters (transit, the time a blood cell passes through the capillary bed, signal decay of regional cerebral blood flow response and ε reflecting the convolution between intravascular and extravascular contribution to the BOLD signal, DCM.Ep); for more information see Friston et al. (2000). Furthermore, we used canonical correlation analysis (CCA; Sui et al., 2012) to identify linear relationships between the two sets of measures (DCM parameters and behavioral performance). The first step was to run CCA on both sets of variables (Set 1, effective connectivity; Set2, cognitive performance in a range of tasks). Linear combinations within each of the sets were defined such that the relationship of these combinations between both sets was maximized. This resulted in pair of significantly correlated canonical variates, i.e., latent variables (V1, connectivity profile; W1, cognitive profile).

We then asked whether the relationship between cognitive performance and DCM parameters was age-dependent using a moderation analysis and/or over and above age. Note that this cross-sectional analysis cannot examine the longitudinal effect of aging per se. Specifically, we constructed a multiple linear model where connectivity profile, age, their interaction term (connectivity profile × age), and covariates of no interest (gender, handedness, level of education, and mean head displacement) were used as independent variables and cognitive profile as a dependent variable. Visual representation of the moderation analysis included scatter plots of connectivity profile versus cognitive profile for three equally sized age groups (1–3rd deciles, young adults; 4–5th deciles, mid-aged adult; and 6–7th deciles, older adults). Because the predictive analysis of age and behavioral performance was based on four network analyses, we applied false-discovery rate (FDR) correction across all multivariate analyses.

To further address the importance of using effective connectivity to characterize age-dependent behavioral variability relative to alternative measures, e.g., functional connectivity, we repeated the multiple linear-regression analysis and CCA using Fisher *z*-transformed correlation coefficients among the network and node time series.

## Results

### Group ICA and network definition

Using group ICA, three independent components were selected with maximal spatial overlap with previously reported SN, DAN, and DMN templates (Shirer et al., 2012). Functional connectivity analysis between all ROIs confirmed that the nodes within each network were highly correlated. In addition, the nodes from DAN and SN were partially correlated, whereas nodes from DAN and DMN were anti-correlated (Fig. 2).

### Effective connectivity analysis

#### Network definition

To define the network structure characterizing between- and within-network connectivity of the three networks (DMN, DAN, and SN), we performed four separate spectral DCM (Friston et al., 2014; Razi et al., 2015) analyses: one between-network and three within-network analyses (within-SN, within-DAN, and within-DMN). The exceedance probability for all models for each network analysis: between-network, within-SN, within-DAN, and within-DMN is shown in Figure 3. We calculated parameter estimates using Bayesian model averaging, where the parameters of each model are weighted by the posterior probability of the model (Hoeting et al., 1999; Penny et al., 2010).

#### Model parameters predict age

**Figure 4.**
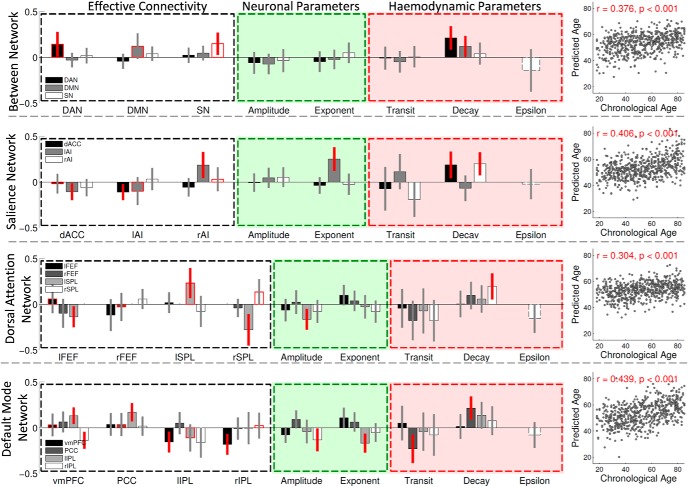
DCM parameters predicting chronological age. Multiple linear-regression coefficients for how well effective connectivity (white), neuronal (green), and hemodynamic (red) DCM parameters predict age from between-network and within-network analyses. DCM parameters having bars with 95% confidence intervals outside zero (red error bars) are considered as significant predictors in the multiple-regression model. Self-inhibitory connections are shown in red outline.

Having derived the weighted means of connectivity parameters using BMA, we asked to what degree the DCM parameters can “predict” participants' age (Fig. 4). For the between-network analysis, we used multiple linear regression and found that ∼20% of age variance in our cohort was predicted (*r* = 0.376, *p* < 0.001) by connectivity parameters. This effect was driven by: (1) increased neural inhibitory self-connections in all networks, and (2) a tendency for increased hemodynamic decay times for all networks (Fig. 4).

Similar effect sizes were observed in the within-network analysis. In particular, for the within-SN analysis, we found that age was independently predicted (*r* = 0.406, *p* < 0.001) by: (1) decreased effective connectivity between dACC and lAI, (2) increased effective connectivity from rAI tolAI, (3) increased exponent of neuronal activity in the dACC, and (4) increased decay times in rAI and dACC (Fig. 4). For the within-DAN analysis, we found that age was predicted (*r* = 0.611, *p* < 0.001) by: (1) tendency for increased neural inhibitory self-connections in all DMN nodes, (2) decreased effective connectivity from lFEF to lSPL and from rSPL to lSPL, (3) decreased amplitude in the left SPL, and (4) increased hemodynamic decay times in the right lSPL (Fig. 4). For the within-DMN, we found that age was predicted (*r* = 0.439, *p* < 0.001) by: (1) decreased effective connectivity from the vmPFC to rIPL and from lIPL to vmPFC, (2) increased effective connectivity from vmPFC and PCC to lIPL, (3) decreased neural amplitude in right IPL, and (4) PCC changes in hemodynamic transit and decay times (Fig. 4).

#### Model parameters and behavioral variability

**Figure 5.**
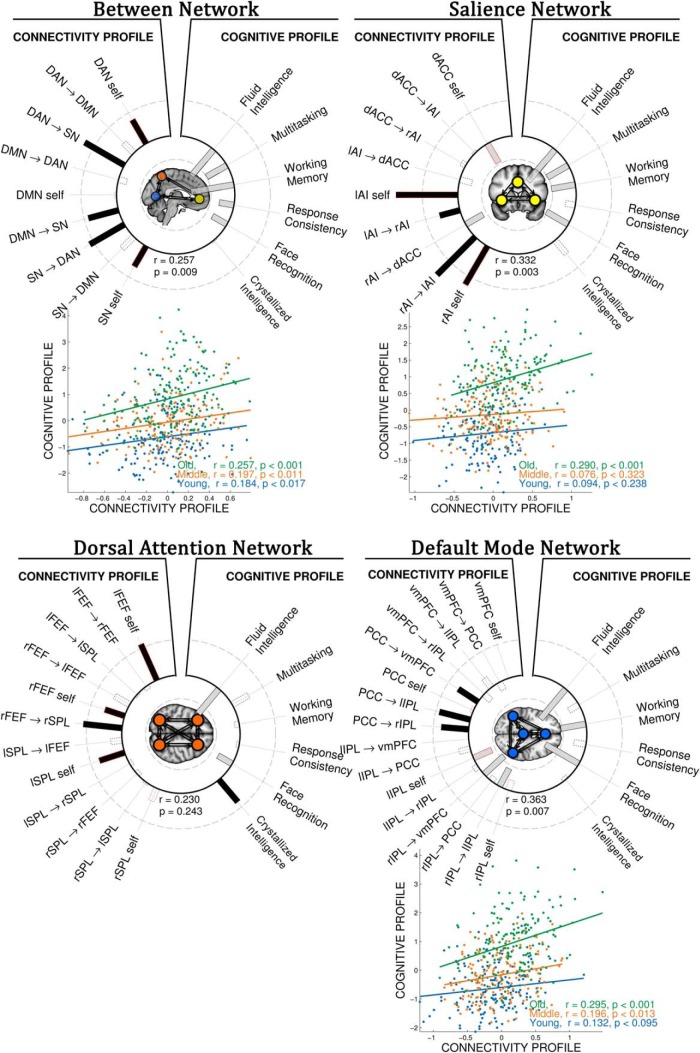
Neural effective connectivity influences cognition, especially in older subjects. Between-network CCA (top, left): heliograph of variate loadings (structural correlations) for the first canonical variate, where the relative size of structure correlations is indicated by the relative length of the bars (dark:positive, white:negative), identifying the statistical relationship between variables of effective connectivity (connectivity profile) and cognitive performance (cognitive profile; *r* = 0.257, *p* = 0.009). Variables with low contribution (*r* < 0.3) are shown in bars with noncontinuous outline. Half-maximum strength of correlation is indicated by the dashed rings (outer is *r* = +0.5, inner is *r* = −0.5). Self-inhibitory connections are shown in red outline. Below heliograph, scatter plot of corresponding bivariate canonical correlation for three age groups. The relationship between connectivity and cognitive profiles is higher for older (formally confirmed by moderation analysis, see “Model parameters and behavioral variability”; Table 1), suggesting that good performance in older adults rely more strongly on a good connectivity profile between networks. Note, higher subject loading value indicates stronger expression of the cognitive profile, i.e., in between-network analysis worse performance in all but one cognitive test. Scatter plot for DAN is not shown given the unreliable relationship between connectivity and cognitive profile.

Using CCA, we asked whether the DCM parameter set was associated with cognitive performance (Fig. 5). For between-network analysis, the corresponding canonical vector identified that poor performance across a range of cognitive tasks was associated with increase in the negative self-inhibition parameter of the DAN and SN networks (i.e., higher performance with more negative self-inhibition) and increased influence of DAN on SN, DMN on SN, and SN on DAN (canonical correlation, *r* = 0.257, *p* < 0.001). The poor cognitive scores included fluid intelligence (Cattell), face processing (Benton Faces), working memory (visual short-term memory) multitasking (Hotel) and response consistency (inverse of response variability of on simple motor task).

For the within-SN analysis, we found that a poor cognitive profile was associated with less negative self-connections within AI nodes, as well as increased influence between right and left AI, and decreased influence of rAI on dACC (*r* = 0.332, *p* = 0.003). For the within-DMN analysis, we observed that poor cognitive performance is associated with increases of PCC influence on other nodes within the DMN, decreased influence of rIPL to vmPFC and lIPF, and more negative lIPL self-connection (*r* = 0.363, *p* = 0.007). For the within-DAN analysis, we found no evidence for a reliable association between effective connectivity parameters within-DMN and cognitive performance (*r* = 0.230, *p* = 0.243), precluding the test for age-related interaction between connectivity and cognitive profiles (i.e., moderation analysis, see the next paragraph).

To further investigate the nature of the relationship between cognitive performance and effective connectivity profiles between networks, within-SN and within-DMN, we conducted a multiple linear regression analysis including age, connectivity, their interaction term (age × connectivity), gender, handedness, level of education, and mean head displacement as independent variable and cognitive performance as dependent variable. The results are shown in Table 1. Specifically, effective connectivity profile (between-networks, within-SN and, within-DMN) was significantly associated with cognitive performance after accounting for the main effect of age and other covariates (*r* = 0.13, *p* < 0.001). Furthermore, the interaction term between age and connectivity profile values (age × connectivity profile) predicted significant variance in cognitive performance, (*r* = 0.08, *p* = 0.034). The direction of the interaction was such that increasing age strengthened the relationship between cognitive and connectivity profiles. Note that age was a continuous variable in the analysis, although for clarity of illustration in Figure 5, we divide the cohort into young, middle and older age groups.

**Table 1.** Regression coefficients of age, connectivity, and their interactions in relation to cognitive performance, where connectivity measures are either effective connectivity (EC) parameters derived from the optimal generative model or functional connectivity (FC) measures

	EC			FC		
	Age	EC	Age × EC	Age	FC	Age × FC
Between network	−0.51***	0.20***	0.08*	−0.62***	0.07†	−0.02^n.s.^
Salience network	−0.64***	0.13***	0.11*	−0.64***	0.08†	0.07^n.s.^
DMN	−0.53***	0.21***	0.12*	−0.61***	0.09†	0.07^n.s.^

*p* value level of significance corrected for multiple comparisons: *** < 0.001; 0.001 < * < FDR; FDR < † < 0.05; n.s., not significant.

### Functional connectivity

The results from the preceding effective connectivity analyses (see Effective connectivity analysis) suggested that it is useful to separate neuronal interactions from hemodynamic signaling, to examine the relationships between age, networks, and cognition. However, it is common to study functional connectivity, based on spatiotemporal covariance of BOLD time series, without separation of neural from neurovascular responses. In this final section, we therefore repeated the analysis of age and behavioral variability in relation to correlation coefficients among the network and nodal time series (after Fisher *z*-transformation).

#### Functional connectivity predicts age

Multiple linear-regression results for each network analysis revealed that functional connectivity correlates with age: *r* = 0.20, *p* < 0.001 (between-network connections), *r* = 0.35, *p* < 0.001 (SN connections), *r* = 0.11, *p* = 0.049 (DAN connections), and *r* = 0.28, *p* < 0.001 (DMN connections; Fig. 2*d*). Individual connections with the highest contribution (and significant at *p* < 0.05, FDR corrected) were consistent with the changes in effective connectivity. In particular, we observed that older age was associated with: (1) reduced functional connectivity between right and left SPL in the DAN, (2) reduced functional connectivity between vmPFC and rIPL and between rIPL and lIPL and increased between PCC and lIPL in the DMN, (3) reduced functional connectivity between dACC and rAI and increased functional connectivity between rAI and lAI increase in the SN, and (4) increased functional connectivity between SN and DMN and reduced functional connectivity between DAN and DMN.

#### Functional connectivity and behavioral variability

**Figure 6.**
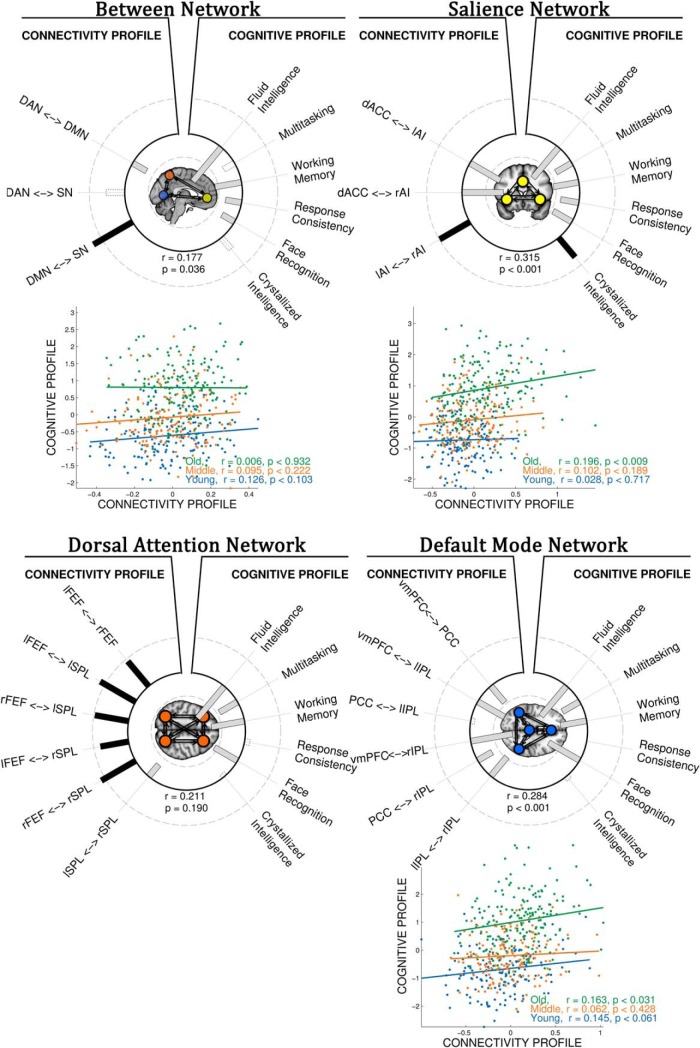
Neurovascular functional connectivity influences cognition, but does not explain age related change. Between-network CCA (top, left), heliograph of variate loadings (structural correlations) for the first canonical variate, where the relative size of structure correlations is indicated by the relative length of the bars (dark:positive, white:negative), identifying the statistical relationship between variables of functional connectivity (connectivity profile) and cognitive performance (cognitive profile; *r* = 0.345, *p* < 0.001). Variables with low contribution (*r* < 0.3) are shown in bars with noncontinuous outline. Half-maximum strength of correlation is indicated by the noncontinuous rings (outer is *r* = +0.5, inner is *r* = −0.5). Self-inhibitory connections are shown in red outline. Below heliograph, scatter plot of corresponding bivariate canonical correlation for three age groups. The relationship between connectivity and cognitive profiles is age-group invariant (tested formally by moderation analysis, see “Functional connectivity and behavioral variability” and Table 1). Note, higher subject loading value indicates stronger expression of the cognitive profile, i.e., in between-network analysis worse performance in all but one cognitive test. Scatter plot for DAN is not shown given the unreliable relationship between connectivity and cognitive profile.

We tested the association between functional connectivity and age related changes in behavioral variability using CCA. Overall functional connectivity CCA (Fig. 6), revealed a similar pattern of connection changes associated with behavioral performance as the effective connectivity CCA (Fig. 5). Specifically, for between-network CCA of functional connectivity, the first canonical vector identified that poor performance across a range of cognitive tasks was associated with increases and decreases in functional connectivity between DMN and the SN and DAN respectively (*r* = 0.345, *p* < 0.001). The increase in undirected connectivity between DMN and SN was in accordance with the increased influence of DMN on SN as revealed by CCA of effective connectivity.

For the within-SN analysis, we found evidence for changes in functional connectivity associated with poorer performance (*r* = 0.315, *p* < 0.001). The increase in functional connectivity between lAI and rAI mirrors the increased influence of rAI on lAI revealed by CCA of effective connectivity. The decreased connectivity between dACC and rAI was likely the result of decreased influence of rAI on dACC, as revealed by CCA of effective connectivity.

For the within-DMN analysis, we observed that decreased functional connectivity within DMN was associated with poor performance (*r* = 0.284, *p* < 0.001). Interestingly, all PPC connections, which showed lowest contributions to functional connectivity CCA, were identified as increased influence of PPC on the other nodes by effective connectivity CCA. For the within-DAN analysis (similarly to the effective connectivity CCA), we found no evidence for a reliable association between functional connectivity parameters within-DAN and cognitive performance (*r* = 0.211 *p* = 0.19).

The critical analysis was of the interaction between age and functional connectivity as a predictor of cognitive performance (Table 1). In contrast to the analyses of effective connectivity above, the main effect of functional connectivity showed a marginal association with cognitive performance. Furthermore, the interaction term between age and functional connectivity profile (age × connectivity profile) was not a significant determinant of cognitive performance (*r* < 0.07, ns). In other words, unlike the CCA of effective connectivity, this CCA of functional connectivity did not confirm the hypothesis that connectivity within and between networks is increasingly important for cognitive performance with older age.

## Discussion

The strength of effective connectivity, within and between large scale functional networks, changed over the healthy adult lifespan, with an increasing influence on cognitive function in older adults. By separating the neural interactions from hemodynamic functions in generative models of the fMRI-BOLD response, we inferred the effects of age on neuronal interactions and the importance of these interactions for cognitive function in healthy adults across the lifespan. We also confirmed significant effects of age on the neurovascular coupling parameters (cf. Tsvetanov et al., 2015).

### Changes in network connectivity relate to chronological age

A consistent tendency for the age-related changes of within- and between-network dynamics was for increased neural time constants of intrinsic inhibitory connections at each node. This may reflect the loss of synaptic gain in local reverberant circuits or reduced disinhibition (Brown and Friston, 2013), such that the activity in a node/network collapses unless an area is extrinsically driven. A corollary is reduced precision and synchronization of action potential timing of excitatory neurons (Buzsáki and Draguhn, 2004; Manseau et al., 2010), consistent with a reduction in effective membrane time constants (Pinotsis et al., 2013) following an age-related change in GABA concentrations (Duarte et al., 2014). This widespread age-related change in intrinsically mediated self-inhibition may be linked to homeostatic regulation of inhibitory activity, important for the generation of spontaneous neural oscillations at rest (Shu et al., 2003) and task-modulated neural inhibition (Muthukumaraswamy et al., 2009).

We also observed decreases in within-network effective connectivity (except for connections to left IPL and from rAI, discussed in the next section). This mirrors the observed effect of age on long-range undirected functional connectivity in large scale networks (Tomasi and Volkow, 2012), network segregation (Geerligs et al., 2015), structural connectomes (Perry et al., 2015), and physiologic coupling as revealed by transcranial magnetic stimulation (Rowe et al., 2006). We suggest that the observed changes in intrinsic inhibition, coupled with reduced within-network connections, promote the loss of network segregation with age.

### Changes in intrinsic connectivity relate to cognition

The connectivity within and between networks explained a large portion of cognitive variability across multiple domains, including fluid intelligence, working memory, response consistency, and face recognition. In particular, we found that faster local inhibitory rate constants (more rapid decay of neural information) were associated with poor cognitive function.

This suggests that brain-wide homeostatic regulation of resting (tonic) inhibitory activity is behaviorally relevant, in line with the association between resting neural inhibition, task-based modulation of inhibition and cognitive performance (Imbrosci and Mittmann, 2011; Heise et al., 2013; Legon et al., 2015). Given the diffuse change of self-inhibitory intrinsic connections, we speculate that the dysregulation in resting inhibition may play a role in a generic decline in central information processing capacity with increasing age (Salthouse, 1996) providing neurobiological support of the inhibition deficit theory of cognitive aging (Hasher and Zacks, 1988). A corollary of this association is that aging would be associated with more diffuse and less specific patterns of task activation, including dedifferentiation and the reduction in hemispheric asymmetry (Park et al., 2010).

Our demonstration that intrinsic interactivity as measured by rs-BOLD activity predicts behavioral variability across multiple cognitive domains has implications beyond aging, including the clinical setting where resting-state effective connectivity may be associated with clinical severity (Hyett et al., 2015). Further investigation of the mechanism of inhibitory processing will be important to establish the mechanisms that link neurophysiology to preserved cognitive function in older life.

### Changes in extrinsic connectivity relate to cognition

Several excitatory connections within and between networks were also identified as behaviorally relevant. Many fMRI studies have identified age-related changes in the strength of connectivity in large scale networks, but few have reported an association of between-network connectivity and age-related cognitive deficits (Baldassarre and Corbetta, 2015). This lack of evidence from functional connectivity studies was also seen from our functional connectivity analysis. This lack of positive evidence may be due to the dependence on covariance among fMRI signal time series, which are confounded by age-related changes in the neurovascular coupling (Logothetis, 2008; Tsvetanov et al., 2015). Here, we dissociated neural from vascular signals, revealing that effective connectivity predicted behavioral performance across multiple tasks, and became an increasingly important determinant of cognition with age. Furthermore, we identified positive associations between connectivity and behavioral variability over and above the effects of aging, in line with our predictions (Kelly et al., 2008).

The multivariate CCA of effective connectivity suggested that poorer cognitive performance is associated with increased influence of the DMN on the SN, coupled with increased influence of the rAI onto the lAI. The right anterior insula is a major node in the salience network, implicated in modulating activity within SN and other networks (Vincent et al., 2008). Therefore, we speculate that the present findings reflect a reassignment of the role of the rAI to compensate and maintain the baseline activity in the lAI, which impedes the ability of the right AI to act as a modulator of between-network interactivity. This in turn results in the increased influence of the DMN on the SN.

Increased coupling of the DMN with the SN may also relate to the reduced connectivity between some of the nodes within the DMN. Indeed, aging reduces within-DMN connectivity and increases connectivity between the DMN and external regions (Chan et al., 2014), leading to reduced segregation of large scale networks. Together, these findings suggest that the aged brain can be characterized by selective vulnerability in excitatory connections within and across large-scale networks, with behavioral consequences that we consider next.

### Connectivity is more important for cognitive function in older age

Effective connectivity, but not functional connectivity, was significantly related to the effect of age on cognition, such that better cognitive performance in older participants relied more strongly on a good connectivity profile between and within large scale networks. We suggest that this is because of an age-related shift in the neural substrates of cognitive functioning (Raz and Rodrigue, 2006). The limited evidence for this hypothesis in previous functional studies may be due to methodological issues, including the confounding effect of age on neurovascular coupling. Our findings suggest that maintaining the resting-state neural connectivity profile becomes increasingly important for maintaining high levels of cognitive function in old age.

Spectral DCM accommodates wide variations in neurovascular coupling, using empirical physiological priors for the hemodynamic response function (Buxton et al., 2004). The estimates of hemodynamic decay suggest dampening of blood flow responsivity with age, consistent with decrease of resting-state fluctuation amplitudes (Tsvetanov et al., 2015). Differences in transit times of the balloon model also suggest reduced blood flow for older adults, consistent with reduced vessel compliance (Flück et al., 2014).

These age-related findings in neurovascular coupling motivate the use of generative models that separate neural and vascular components of the fMRI signal. Failure to separate these signal components will confound fMRI studies of the effects of age on activation and functional connectivity (Tsvetanov et al., 2015). Indeed, the vascular changes identified by spectral DCM are in line with independent but more complex methods (Liu et al., 2013). We used a power-law model of the coupled dynamics of neuronal populations to generate complex cross-spectra among measured BOLD responses. This is distinct from “stochastic DCM” methods (Li et al., 2011) as it overcomes the difficulty of estimating random fluctuations in neural states. We observed weak age-related decreases of the amplitude of endogenous fluctuations driving neuronal populations in dACC and bilateral IPL, perhaps due to loss of neural density. In addition, there was marginally significant age-related increase of the exponent of neural fluctuations, which may suggest that as people get older the exponent of scale-free fluctuations decreases. Further work is required to show whether this reflects a change in the rapid switching between transient modes (Woolrich et al., 2013).

### Limitations and future considerations

Our generative models or neuronal dynamics are linear systems and precluded state-dependent changes in effective connectivity, i.e., we treated the resting-state time series as a stationary process (Razi et al., 2015). This model of time-invariant effective connectivity in addition to the model space selection provides a limited repertoire of dynamical behavior, albeit one that can explain a significant proportion of variance in aging and cognition. In addition, we used a single-state model of each node and network. Future studies may provide further insights by encompassing nonlinear interactions between regions (Goulden et al., 2014), or multistate variables for inhibitory-excitatory subpopulations (Marreiros et al., 2008).

Unlike most cross-sectional neurocognitive studies, our cohort was drawn from a population-based epidemiological study. However, even though the population sampling method for this study sought to minimize age-based cohort effects, future studies would be strengthened by longitudinal analysis to identify the mediators of the rates of change and the determinants of cognitive resilience.

### Concluding remarks

Using generative models to dissociate neural from vascular components of the fMRI signal, we discovered behaviorally relevant and age-dependent differences in resting-state effective connectivity. These were manifest both within and between large-scale networks, and were associated with faster decay of local neural activity. Maintaining resting-state connectivity profile was increasingly relevant for older adults to maintain cognitive function across many domains. We propose that preventive and interventional strategies that target such connectivity will promote the wellbeing of individuals and the “mental wealth of nations” (Beddington et al., 2008).
